# Association between dosing of spironolactone and outcomes in heart failure with preserved ejection fraction patients combined with chronic kidney disease------Balance of efficacy and risk

**DOI:** 10.3389/fphar.2023.1084442

**Published:** 2023-01-27

**Authors:** Jun-Feng Li, Xiang Qu, Zhan Gao, Chang-Xi Chen, Feng-Yu Zhang, Ling Cheng, Xi Zhou, Hao Zhou

**Affiliations:** Department of Cardiology, The First Affiliated Hospital of Wenzhou Medical University, Wenzhou, Zhejiang, China

**Keywords:** heart failure with preserved ejection fraction, chronic kidney disease, spironolactone, dose, all-cause death, hospitalization for heart failure

## Abstract

**Aims:** Few studies have compared the association between dosing of spironolactone and outcomes in patients with heart failure with preserved ejection fraction (HFpEF), and whether spironolactone dose could significantly affect the prognosis of HFpEF patients combined with chronic kidney disease (CKD) remains unclear. Our aim was to directly compare ‘high vs. low’ doses of spironolactone in an attempt to find a benefit-risk-balanced point, and infer an adequate dose for HFpEF with CKD patients.

**Methods:** Overall, 4,321 symptomatic heart failure inpatients were initially screened from January 2013 to December 2019, and all enrolled patients were followed-up for 36 months; After including patients who meet the diagnostic criteria of HFpEF and CKD with ejection fraction > 45% and estimated glomerular filtration rate (eGFR) < 60 ml/min/1.73 m^2^, a total of 387 patients was selected. Primary outcome was a composite of all-cause death, heart failure (HF) hospitalization and non-fatal stroke. The key safety outcome was hyperkalemia rates during the follow-up period.

**Results:** The primary outcome event rates in patients with or without spironolactone were 12.74 and 21.45 per 100 person-years, respectively. Compared with patients not taking spironolactone, the adjusted hazard ratio (HR) [95% confidence interval (CI)] was 0.55 (0.38–0.79) with spironolactone group for primary outcomes. After grouped by the daily dose of spironolactone, low-dose group (≤ 40 mg) was associated with lower relative risk for the primary efficacy outcome [adjusted HR (95% CI) was 0.43 (0.23–0.81), 0.50 (0.33–0.76) and 0.74 (0.36–2.79) with < 40 mg, 40 mg and >40 mg, respectively]. During 3-year follow-up, the risk for hyperkalemia was amplified in the higher dose group (>40 mg) while showed no significant difference compared with low dose group (*p* = 0.425).

**Conclusion:** HFpEF with CKD patients using spironolactone had lower risk of adverse cardiovascular outcomes. And the use of low-dose spironolactone (≤ 40 mg) showed the best efficacy and safety, therefore we may recommend ≤ 40 mg as the optimal initial dose for these patients. However, this was a relatively small sample size, retrospective study, and further adequately powered randomized trials are needed to verify these results.

## Introduction

Heart failure with preserved ejection fraction (HFpEF) is a clinical syndrome characterized by patients presenting with symptoms and signs of heart failure but with normal or near-normal left ventricular ejection fraction (Gladden et al., 2014). Among heart failure patients, the estimated incidence of HFpEF is approximately 50%, and the studies have suggested that the proportion of HFpEF is increasing (Steinberg et al., 2012). Owing to the rising prevalence and limited treatment options, HFpEF becomes a major clinical and public health problem (Gerber et al., 2015). In the past 30 years, the drug treatment of heart failure has been continuously improved. However, the development of drug therapy for HFpEF has failed to meet expectations and several large clinical trials have shown neutral results (Yusuf et al., 2003; Cleland et al., 2006; Solomon et al., 2019). While recently, the EMPEROR-Preserved (Empagliflozin in Heart Failure with a Preserved Ejection Fraction) and DELIVER (Dapagliflozin in Heart Failure with Mildly Reduced or Preserved Ejection Fraction) studies confirmed the benefit of sodium-dependent glucose transporters two inhibitor (SGLT2i) in reducing the combined risk of cardiovascular death or hospitalization for heart failure in HFpEF patients (Anker et al., 2021; Solomon et al., 2022). Similarly, the TOPCAT (Treatment of Preserved Cardiac Function Heart Failure With an Aldosterone Antagonist) trial concluded that the spironolactone did not reach the primary composite endpoint of cardiovascular death, aborted cardiac arrest, or hospitalization for the management of heart failure (HR = 0.89) in HFpEF patients, but did reduce HF hospitalizations (HR = 0.83) (Pitt et al., 2014). While, *post hoc* analysis which enrolled the America patients showed significant efficacy of spironolactone in reducing rates of the primary composite, HF hospitalization, and cardiovascular death (Pfeffer et al., 2015). Based on these analyses, the guideline suggested a IIB recommendation for using aldosterone receptor antagonist, like spironolactone, in appropriately selected patients with symptomatic HFpEF (Yancy et al., 2017). HFpEF patients are more commonly combined with CKD, and are associated with poorer prognostic outcomes (Borlaug, 2020). Using spironolactone can improve the prognosis of HFpEF patients with advanced CKD. However, with increased adverse events such as hyperkalemia, the closer laboratory surveillance of serum potassium is needed (Beldhuis et al., 2019). Moreover, due to the effects of spironolactone on renal function and hyperkalemia, the exact dosage adjustment in these patients remains unclear. The initial administration dose in TOPCAT was 15 mg/day, and within the next 4 months, the investigator would increase the dose to a maximum 45 mg/day depend on patients’ safety parameters (Pitt et al., 2014). However, after TOPCAT, there was still no guideline make a recommendation on the optimal dose of spironolactone for HFpEF, especially combined with CKD patients. Ferreira et al. conducted a *post hoc* analysis of spironolactone dose with patients from “TOPCAT-Americas” and suggested that for elderly HFpEF patients with high serum potassium levels and impaired renal function, < 25 mg (around 15 mg/day–20 mg/day) of spironolactone might be a better choice instead of stopping treatment (Ferreira et al., 2020). While this analysis only focused on the high-risk groups of HFpEF (eg, elder with renal dysfunction and hyperkalemia) rather than HFpEF with CKD patients, and did not directly compare the effect and safety between dose categories, therefore a more specific spironolactone dose cannot be determined. In addition, the TOPCAT trial and its *post hoc* analysis excluded patients with eGFR <30 ml/min/1.73 m^2^, and our study targeted the full range of eGFR in CKD patients, including dialysis patients.

Thus, in this real-world cohort of HFpEF with CKD patients, we explored the association between the dose of spironolactone and outcomes in these patients. Additionally, we aimed to deduce an efficacy-risk-balanced dose through a direct comparison of different dose categories in patients with HFpEF and CKD.

## Methods

### Study design and population

In this single-center, clinical, retrospective cohort study, 4,321 inpatients based on diagnosis of HF according to the International Classification of Diseases (ICD) codes were initially screened from January 2013 to December 2019, the clinical data and patients’ variables were recorded using a web-based Electronic-Medical-Records System (EMR) in hospital database, based on inpatient and outpatient clinic visit results. And patients who meet diagnostic criteria for HFpEF and CKD will be ultimately selected. The study protocol was approved by the local ethics committee.

### Definition of CKD

According the Chronic Kidney Disease Epidemiology Collaboration equation (CKD-EPI), we obtained patients’ eGFR by calculating their creatinine level during hospitalization, and the eGFR was used to assess the patients’ renal function. CKD was defined as estimated GFR <60 mL/min per 1.73 m^2^. To avoid the inclusion of patients with AKI, we dynamically followed up the patients’ renal function for the next two months after including patients with eGFR<60 mL/min per 1.73 m^2^ measured at the first time of admission. Patients whose renal function recovered after discharge and eGFR > 60 mL/min per 1.73 m^2^ would be excluded. Severe renal dysfunction patients (defined as dialysis and eGFR <30 mL/min per 1.73 m^2^) will not be excluded.

### Definition of HFpEF

To date, the diagnosis of HFpEF remains challenging. Owing to its normal ejection fraction (EF) and non-specific signs and symptoms, physicians often do not discriminate well between HFpEF and other clinical conditions (Ferreira et al., 2020). There is no recommended consensus diagnostic criteria for the HFpEF, and clinical trials have used variable definitions of HFpEF, mainly focus on the cut-off of left ventricle ejection fraction (LVEF) (eg, LVEF ≥ 40%, 45%, or 50%). In our research, we integrated the diagnostic criteria of HFpEF in latest European Society of Cardiology (ESC) (Ponikowski et al., 2016) and American Heart Association (AHA) guidelines (Yancy et al., 2013; Heidenreich et al., 2022), and eligible requirements of four large HFpEF clinical trials (I-Preserve, TOPCAT, PARAGON-HF, EMPEROR-Preserve) (Massie et al., 2008; Pitt et al., 2014; Solomon et al., 2019; Anker et al., 2021). In short, patients aged more than 18 years old if they had i) Concurrent at least one symptom and sign of heart failure (eg, symptoms for shortness of breath, orthopnea or paroxysmal nocturnal dyspnea; signs for elevated jugular venous pressure or positive hepatic jugular reflux sign), ii) LVEF ≥ 45% measured by echocardiography and without a history of overtly reduced LVEF (< 45%), iii) New York Heart Association (NYHA) class II-IV, iv) Elevated left ventricle filling pressure: brain natriuretic peptide (BNP) level ≥ 100 pg/ml or BNP ≥105 pg/ml for atrial fibrillation (AF) patients, v) Abnormal cardic structure: left ventricle mass index (LVMI) ≥115 g/m^2^ (male); ≥ 95 g/m^2^ (female). Patients who met these criteria will be diagnosed as HFpEF.

Key exclusion criteria were any of the following conditions: serum potassium level at first admission > 5.5 mmol/L; taking certain medications known to cause kidney damage such as hyperkalemia (e.g., non-steroidal anti-inflammatory drugs (NSAIDs) such as ibuprofen and naproxen, as well as some traditional Chinese medicine); combined with malignant disease or severe systemic illness with life expectancy of < 3 years; Among 4,321 HF patients, after screening patients with an identified eGFR and who meet the criteria of HFpEF, a total of 387 HFpEF with CKD patients were finally selected, including 31 dialysis patients (8.01%).

### Follow-up and outcome measures

All selected participants were asked for returning for a routine outpatient follow-up at 1 month and then each year after discharge. Our research residents were able to access into the EMR which can provide the outcome HF hospitalization and other additional comorbidities or by telephone call to collect the follow-up data. The primary outcome was a composite of all-cause death, HF hospitalization and non-fatal stroke. The primary safety outcome was hyperkalemia which considered as serum potassium > 5.5 mmol/L during the follow-up period. Patient follow-up was censored at the time of death. The median follow-up duration was 2.7 years.

### Statistical analysis

Patients who met the study selection criteria were divided into two categories according to whether they were treated with spironolactone. Furthermore, the patients in spironolactone group were also stratified into three groups according to daily treating dose (< 40 mg; 40 mg; > 40 mg), we refer to < 40 mg and 40 mg collectively as low-dose group, and > 40 mg as the high-dose group. Consequently, the associations with outcomes of i) treating with spironolactone or not; and of ii) the different doses of spironolactone were assessed.

Baseline characteristics of patients with or without spironolactone were presented as counts and proportions (%), as mean ± standard deviations (SDs), or as median and interquartile ranges (IQR). The groups were compared using student *t*-test or Mann-Whitney U test for continuous variables, and chi-square test were used for categorical variables.

The prespecified primary outcome and its components (all-cause death, HF hospitalization and stroke) analysis was a time-to-event analysis with the use of Kaplan–Meier survival curves and unadjusted log-rank test. To evaluate the effect of spironolactone and the relationship between the different dose categories and outcomes, crude and multivariate adjusted Cox proportional hazard models were performed. For the multivariate adjustment, we included the following variables: age, sex, body mass index (BMI), smoking, alcohol, NYHA III/IV, hypertension, diabetes, systolic blood pressure, heart rate, myocardial infarction, coronary artery disease, atrial fibrillation, stroke, potassium, creatintine, eGFR, LVEF, use of angiotensin-converting–enzyme inhibitors (ACEI)/angiotensin-II–receptor blockers (ARB), use of beta-blockers, use of diuretics, use of statin. Hazard ratios (HRs), 95% confidence intervals (CIs), and *p* values were calculated fitting the Cox models. Additionally, we assessed the association of different eGFR categories and outcomes using the unadjusted Cox regression model, and to obtain whether the spironolactone effect depend on eGFR, we conducted an interaction term between primary outcome and categorical eGFR in its Cox model. At last, values obtained from efficacy and safety outcome were used to plot the relationship among treatment, outcome, and dose of spironolactone.

All analyses were conducted with the use of SPSS software, version 25 (IBM Corporation, United States). The calculations were performed with Medcalc 12.9 statistical software (Mariakerke, Belgium). A value of *p* < 0.05 in the 2-tailed test was considered to indicate statistical significance.

## Results

**FIGURE 1:**
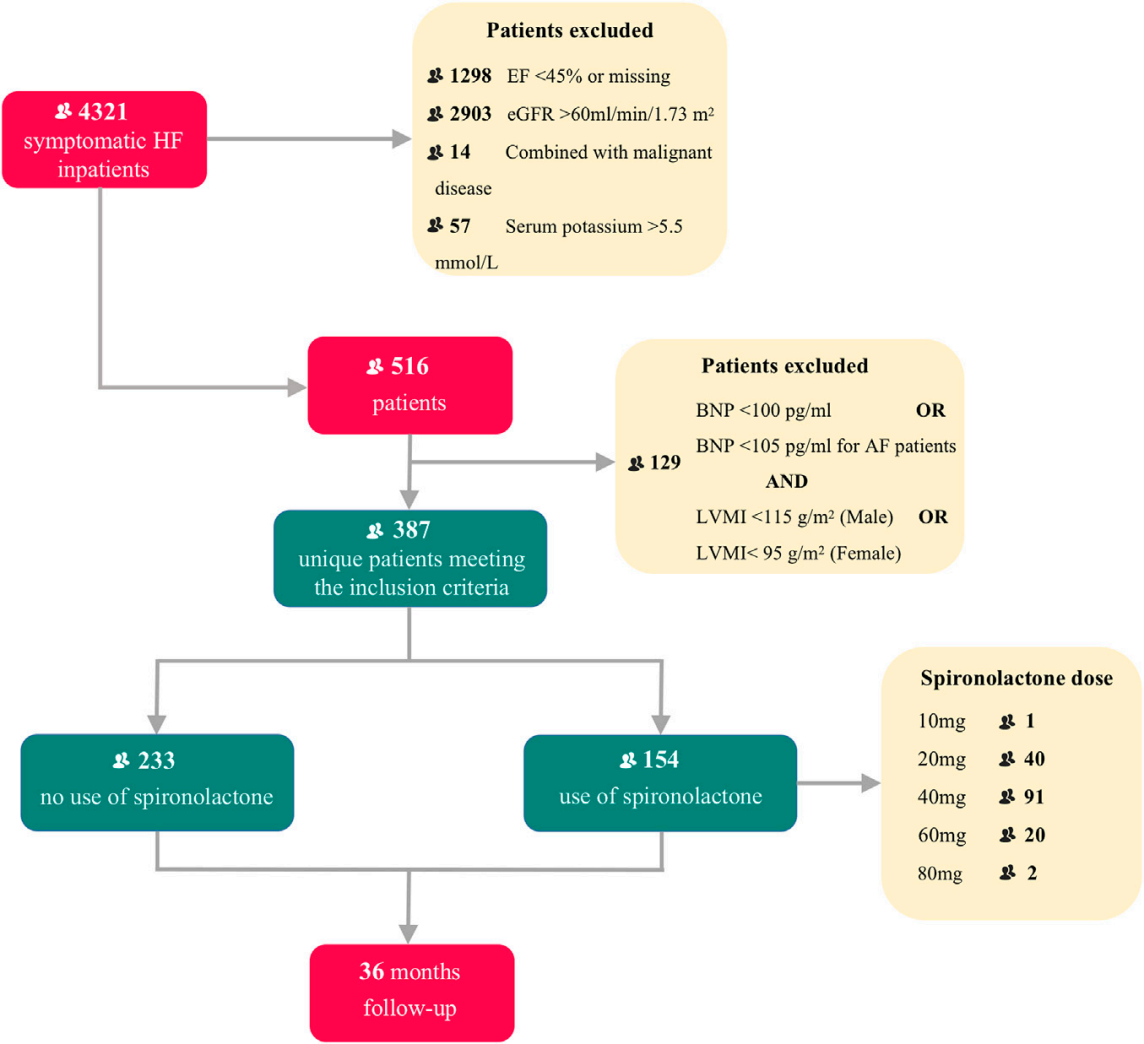
Title: Flow chart of patient enrollment and follow-up Legend: Flow chart describing patients selection. HF, heart failure; EF, ejection fraction; eGFR, estimated glomerular filtration rate; BNP, brain natriuretic peptide; AF, atrial fibrillation; LVMI, left ventricle mass index.

From 1 January 2013 to 31 December 2019, 4,321 inpatients were recorded in our initial enrollment. After selection of the final diagnostic criteria, 387 HFpEF patients (ejection fraction ≥ 45%) combined with CKD were finally considered for this analysis, including 154 patients (60.2%) taking spironolactone and 233 patients (39.8%) not taking spironolactone. Study profile is presented in Figure 1.

### Patient characteristics

Baseline characteristics of the overall population were shown in Table 1. Compared with those not taking spironolactone, patients in spironolactone group were older, less likely to have diabetes, had higher ACEI/ARB and diuretics requirement, lower LVEF, and higher heart rates. In contrast, patients not using the spironolactone had worse eGFR, and nearly half of them were in the lowest eGFR category (severe renal dysfunction).

**TABLE 1:** Baseline characteristics of study patients with chronic kidney disease.

	All enrolled patients		
Characteristics	Spironolactone (-) *n* = 233	Spironolactone (+) *n* = 154	*p*-value
Demographics			
Age, years	74.1 ± 10.4	76.2 ± 9.3	0.046
Male, *n* (%)	131 (56.2)	83 (53.9)	0.652
Body mass index, kg/m^2^	24.1 ± 13.6	23.0 ± 3.8	0.315
Smoking status, *n* (%)			
Never	176 (75.5)	111 (72.1)	
Former	57 (24.5)	43 (27.9)	0.447
Alcohol drinks, *n* (%)			
Never	196 (84.1)	136 (88.3)	
Former	37 (15.9)	18 (11.7)	0.248
NYHA functional classifications, *n* (%)			
II	49 (21.0)	34 (22.1)	0.806
III	143 (61.4)	102 (66.2)	0.332
IV	41 (17.6)	18 (11.7)	0.113
III/IV	184 (79.0)	120 (77.9)	0.806
Hypertension, *n* (%)	193 (82.8)	124 (82.5)	0.563
Diabetes, *n* (%)	106 (45.5)	45 (29.2)	0.001
Systolic blood pressure, mmHg	139.7 ± 30.2	136.4 ± 26.0	0.268
Diastolic blood pressure, mmHg	71.5 ± 15.9	73.4 ± 15.1	0.233
Heart rate, beats/min	76.4 ± 19.8	81.9 ± 23.4	0.014
History of cardiovascular events, *n* (%)			
Myocardial infarction	9 (3.9)	2 (1.3)	0.241
Coronary artery disease	50 (21.5)	26 (16.9)	0.267
Atrial fibrillation	68 (29.2)	54 (35.1)	0.223
Stroke	29 (12.4)	16 (10.4)	0.537
Laboratory variables			
BNP, pg/mL	931.0 (484.0–1798.0)	1,119.0 (653.5–2,357.5)	0.002
Serum potassium, mmoI/L	4.1 (3.7–4.5)	4.0 (3.6–4.3)	0.085
Creatinine, μmoI/L	171.0 (127.0–317.0)	142.5 (118.0–171.0)	<0.001
Estimated GFR, ml/min per 1.73m^2^	29.3 ± 16.6	36.6 ± 12.5	<0.001
45 ≤ eGFR<60	55 (23.6)	47 (30.5)	0.131
30 ≤ eGFR<45	60 (25.8)	59 (38.3)	0.009
eGFR < 30	118 (50.6)	48 (31.2)	<0.001
Echocardiographic data			
LVEF, (%)	57.5 ± 8.5	54.9 ± 7.7	0.002
Medications, *n* (%)			
ACEI/ARB	91 (39.1)	85 (55.2)	0.002
Dose of spironolactone, *n* (%)			
< 40 mg	no	41 (26.6)	
40 mg	no	91 (59.1)	
> 40 mg	no	22 (14.3)	
Beta-blockers	107 (45.9)	86 (55.8)	0.056
Diuretics	128 (54.9)	143 (92.9)	<0.001
Statin	181 (77.7)	126 (81.8)	0.325

Values are given as number of participants, percent, or mean (standard deviation). NYHA, new york heart association; BNP, brain natriuretic peptide; eGFR, estimated glomerular filtration rate; LVEF, left ventricle ejection fraction; ACEI, angiotensin-converting enzyme inhibitor; ARB, angiotensin receptor blocker.

Table 2 summarize patient characteristics according to the daily dose of spironolactone. Patients receiving less than 40 mg dose of spironolactone had lower prevalence of diabetes, higher diastolic blood pressure, lower creatinine level, higher eGFR, lower LVEF, and more frequent use of ACEI/ARB and beta-blockers.

**TABLE 2:** Baseline characteristics of study patients categorized according to the dose of spironolactone.

	Dose of spironolactone				
Characteristics	No use *n* = 233	<40 mg *n* = 41	40 mg *n* = 91	>40 mg *n* = 22	*p*-value
Demographics					
Age, years	74.1 ± 10.4	76.6 ± 10.0	76.4 ± 8.9	74.3 ± 9.9	0.183
Male, *n* (%)	131 (56.2)	24 (58.5)	48 (52.7)	11 (50.0)	0.769
Body mass index, kg/m^2^	24.1 ± 13.6	23.8 ± 3.4	22.8 ± 4.0	22.7 ± 3.7	0.754
Smoking status, *n* (%)					
Never	176 (75.5)	25 (61.0)	71 (78.0)	15 (68.2)	
Former	57 (24.5)	16 (39.0)	20 (22.0)	7 (31.8)	0.167
Alcohol drinks, *n* (%)					
Never	196 (84.1)	34 (82.9)	80 (87.9)	22 (100.0)	
Former	37 (15.9)	7 (17.1)	11 (12.1)	0 (0.0)	0.188
NYHA functional classifications, *n* (%)					
II	49 (21.0)	9 (22.0)	21 (23.1)	4 (18.2)	0.958
III	143 (61.4)	29 (79.7)	58 (63.7)	15 (68.2)	0.664
IV	41 (17.6)	3 (7.3)	12 (13.2)	3 (13.6)	0.343
III/IV	184 (79.0)	32 (78.0)	70 (76.9)	18 (81.8)	0.958
Hypertension, *n* (%)	193 (82.8)	30 (73.2)	74 (81.3)	20 (90.9)	0.325
Diabetes, *n* (%)	106 (45.5)	5 (12.2)	32 (35.2)	8 (36.4)	0.001
Systolic blood pressure, mmHg	139.7 ± 30.2	136.2 ± 26.1	135.8 ± 24.0	139.1 ± 34.3	0.693
Diastolic blood pressure, mmHg	71.5 ± 15.9	78.3 ± 15.0	70.4 ± 14.4	76.6 ± 15.9	0.020
Heart rate, beats/min	76.4 ± 19.8	78.8 ± 21.6	82.5 ± 24.2	85.3 ± 23.9	0.057
History of cardiovascular events, *n* (%)					
Myocardial infarction	9 (3.9)	0 (0.0)	1 (1.1)	1 (4.5)	0.322
Coronary artery disease	50 (21.5)	10 (24.4)	12 (13.2)	4 (18.2)	0.320
Atrial fibrillation	68 (29.2)	18 (43.9)	32 (35.2)	4 (18.2)	0.118
Stroke	29 (12.4)	4 (9.8)	8 (8.8)	4 (18.2)	0.580
Laboratory variables					
BNP, pg/mL	931.0 (484.0–1798.0)	883.0 (513.0–1743.5)	1,396.0 (726.0–2,649.0)	1,119.0 (772.8–2066.8)	0.002
Serum potassium, mmoI/L	4.1 (3.7–4.5)	4.0 (3.5–4.2)	4.1 (3.6–4.4)	3.9 (3.7–4.2)	0.209
Creatinine, μmoI/L	171.0 (127.0–317.0)	125.0 (110.0–150.5)	150.0 (125.0–184.0)	150.0 (119.0–210.3)	<0.001
Estimated GFR, ml/min per 1.73m^2^	29.3 ± 16.6	42.8 ± 10.7	34.6 ± 11.9	33.1 ± 14.5	<0.001
45 ≤ eGFR<60	55 (23.6)	22 (53.7)	19 (20.9)	6 (27.3)	<0.001
30 ≤ eGFR<45	60 (25.8)	12 (29.3)	39 (42.9)	8 (36.4)	0.025
eGFR< 30	118 (50.6)	7 (17.1)	33 (36.3)	8 (36.4)	<0.001
Echocardiographic data					
LVEF, (%)	57.5 ± 8.5	53.7 ± 7.9	55.0 ± 7.5	56.9 ± 8.4	0.009
Medications, *n* (%)					
ACEI/ARB	91 (39.1)	28 (68.3)	46 (50.5)	11 (50.0)	0.003
Beta-blockers	107 (45.9)	28 (68.3)	49 (53.8)	9 (40.9)	0.040
Diuretics	128 (54.9)	39 (95.1)	83 (91.7)	21 (95.5)	<0.001
Statin	181 (77.7)	35 (85.4)	74 (81.3)	17 (77.3)	0.666

Values are given as number of participants, percent, or mean (standard deviation). Abbreviations as in Table 1.

## Outcome analysis

### Effect of spironolactone on outcomes

**FIGURE 2:**
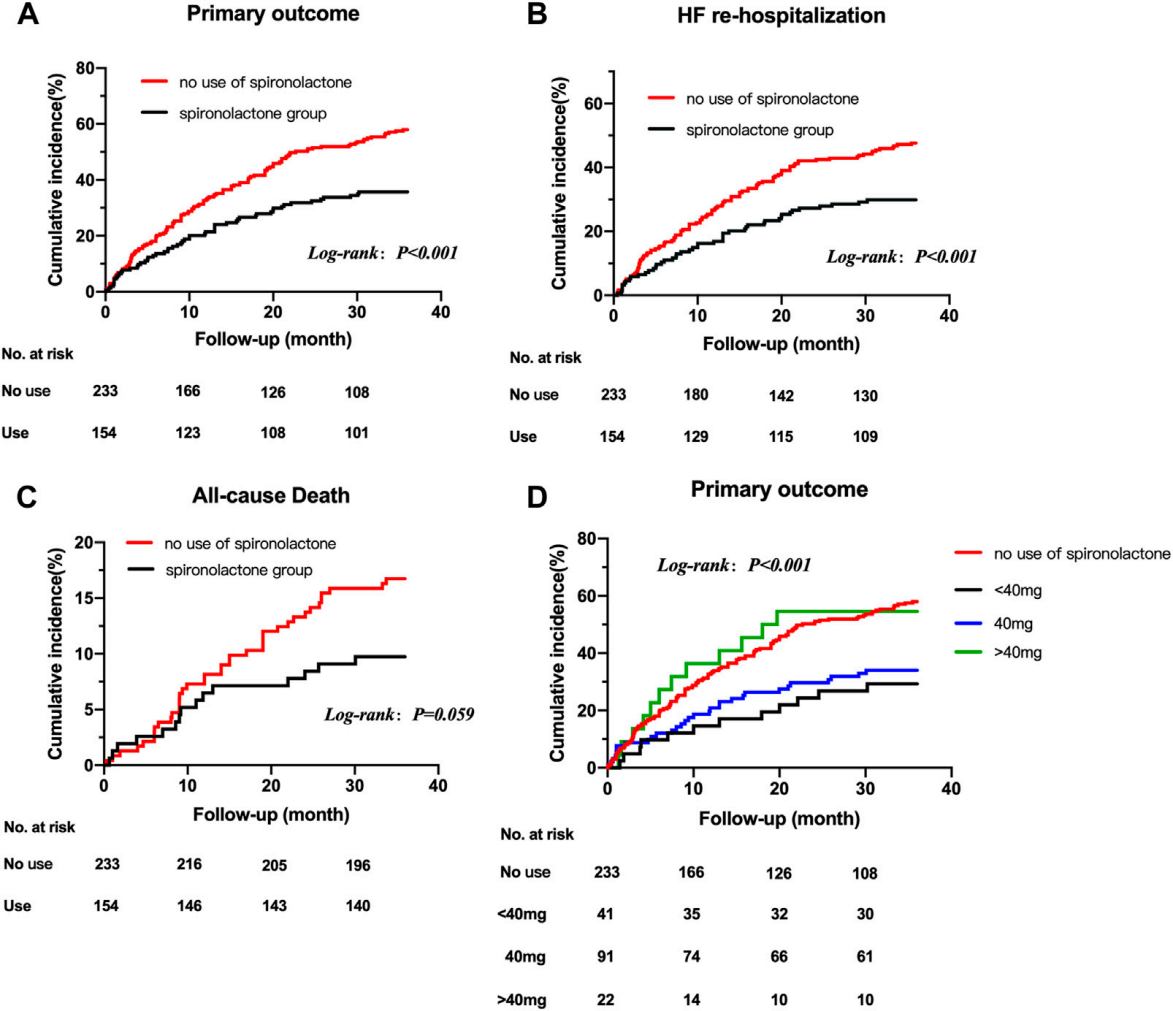
Title: Kaplan-Meier survival curves for outcomes in HFpEF patients with CKD taking and not taking spironolactone Legend: Kaplan-Meier survival curves for outcomes in HFpEF patients with CKD taking and not taking spironolactone. Rates of freedom from primary outcome events **(A)** HF re-hospitalization **(B)** all-cause death **(C)** and primary outcome events grouped by dose of spironolactone **(D)** Cumulative incidence of stroke not shown because of the very low number of observations. HFpEF, heart failure with preserved ejection; CKD, chronic kidney disease.

The mean follow-up period was 2.7 years. 190 patients underwent at least one confirmed primary event during follow-up. The incidence of primary endpoint in patients taking and not taking spironolactone was 12.74 (95% CI: 7.47–18.00) and 21.45 (95% CI: 16.18–26.72) per 100 patient-years (Table 3). Compared with no use, the risks of primary outcome and HF hospitalization were significantly lower in patients taking spironolactone [unadjusted HR (95% CI) for primary outcome: 0.53 (0.39–0.73), *p* < 0.001, Figure 2A; unadjusted HR (95% CI) for HF hospitalization: 0.56 (0.40–0.79), *p* = 0.001; Figure 2B; unadjusted HR (95%CI) for all-cause death: 0.57 (0.31–1.03), p=0.062; Figure 2C]. Consistently, after adjustment for variables that were significantly different in baseline characteristic, use of spironolactone was associated with lower risk of primary outcome or HF hospitalization [adjusted HR (95% CI) for primary outcome: 0.55 (0.38–0.79), *p* = 0.001; adjusted HR (95% CI) for HF hospitalization: 0.63 (0.42–0.93), *p* = 0.018].

**TABLE 3:** Primary outcomes events in patients with chronic kidney disease taking and not taking spironolactone^a^.

	Spironolactone (+) *n* = 154	Spironolactone (-) *n* = 233	*p*-Value
Event			
Primary outcome events^b^			
No. of patients	55	135	
Event rate, per 100 person-years (95% CI)	12.74 (7.47–18.00)	21.45 (16.18–26.72)	
Unadjusted HR (95% CI)	0.53 (0.39–0.73)	Ref	<0.001
*Adjusted HR (95% CI)	0.55 (0.38–0.79)	Ref	0.001
All cause death			
No. of patients	15	39	
Event rate, per 100 person-years	3.47 (0.58–6.37)	6.20 (3.10–9.29)	
Unadjusted HR (95% CI)	0.57 (0.31–1.03)	Ref	0.062
*Adjusted HR (95% CI)	0.57 (0.29–1.14)	Ref	0.119
Re-hospitalization for heart failure			
No. of patients	46	111	
Event rate, per 100 person-years	10.65 (5.78–15.52)	17.64 (12.74–22.53)	
Unadjusted HR (95% CI)	0.56 (0.40–0.79)	Ref	0.001
*Adjusted HR (95% CI)	0.63 (0.42–0.93)	Ref	0.018
Stroke			
No. of patients	1	7	
Event rate, per 100 person-years	0.23 (0.20–0.26)	1.11 (1.07–1.16)	
Unadjusted HR (95% CI)	0.21 (0.03–1.74)	Ref	0.150
*Adjusted HR (95% CI)	0.05 (0.01–1.54)	Ref	0.086

^a^ Data are presented as number or hazard ratio (95% CI).

^b^ The primary outcome was a composite of all-cause death, hospitalization for heart failure or stroke. HR: hazard ratio; CI: confidence interval; Ref.: reference. *= Adjusted for covariates including ages, sex, body mass index, smoking, alcohol, NYHA III/IV, hypertension, diabetes; SBP, heart rate, myocardial infarction, coronary artery disease, atrial fibrillation, stroke, potassium, creatintine, eGFR, LVEF, ACEI/ARB, beta-blockers, diuretics, statin.

### Association between different dose of spironolactone and outcomes

After grouped by the daily dose of spironolactone (< 40 mg, 40 mg and > 40 mg), there were 233 patients in no use of spironolactone group, 41 patients in < 40 mg group, 91 patients in 40 mg group and 22 patients in > 40 mg group. Compared with no use, low-dose category (< 40 mg and 40 mg) of spironolactone was associated with lower risk of primary outcome and HF hospitalization. With no use as reference, the unadjusted HR (95% CI) was 0.41 (0.23–0.73) with < 40 mg group, 0.50 (0.34–0.74) with 40 mg group for primary outcome; 0.48 (0.26–0.89) with < 40 mg group, 0.51 (0.33–0.79) with 40 mg group for HF hospitalization. While, high-dose category (> 40 mg) of spironolactone showed no significant difference compared with no use group [unadjusted HR (95% CI) for primary outcome: 0.99 (0.55–1.79)] (Table 4 A, Figure 2D). With high-dose group (> 40 mg) as reference, < 40 mg and 40 mg group was associated with a 59% and 50% lower risk of primary outcome (Table 4 B) [unadjusted HR (95% CI) with < 40 mg group: 0.41 (0.18–0.91); unadjusted HR (95% CI) with 40 mg group: 0.50 (0.26–0.98)]. After extensive adjustments, the relationship remained as before, the risk of primary outcome was still lower in patients taking low-dose (< 40 mg and 40 mg) spironolactone compared with patients not taking spironolactone [adjusted HR (95% CI) for primary outcome with < 40 mg group: 0.43 (0.23–0.81); adjusted HR (95% CI) for primary outcome with 40 mg group: 0.50 (0.33–0.76)].

**TABLE 4:** Primary outcomes events in patients categorized according to the dose of spironolactone (Risk estimates calculated using no-use as reference)^a^.

	Dose of spironolactone			
Events	No use *n* = 233	< 40 mg *n* = 41	40 mg *n* = 91	> 40 mg *n* = 22
Primary outcome events^b^				
No. of patients	135	12	31	12
Event rate, per 100 person-years	21.45 (16.18–26.72)	10.16 (0.91–19.41)	12.14 (5.43–18.85)	20.52 (3.64–37.40)
Unadjusted HR (95% CI)	Ref	**0.41 (0.23–0.73)**	**0.50 (0.34–0.74)**	0.99 (0.55–1.79)
*Adjusted HR (95% CI)	Ref	**0.43 (0.23–0.81)**	**0.50 (0.33–0.76)**	1.18 (0.36–2.79)
All cause death				
No. of patients	39	2	10	3
Event rate, per 100 person-years	6.20 (3.10–9.29)	1.69 (1.40–2.02)	3.92 (3.71–4.13)	5.13 (4.19–6.16)
Unadjusted HR (95% CI)	Ref	0.28 (0.07–1.15)	0.64 (0.32–1.28)	0.84 (0.26–2.71)
*Adjusted HR (95% CI)	Ref	0.19 (0.02–1.68)	0.55 (0.18–1.72)	0.56 (0.06–5.59)
Re-hospitalization for heart factors				
No. of patients	111	11	25	10
Event rate, per 100 person-years	17.64 (12.74–22.53)	9.32 (0.42–18.21)	9.79 (3.69–15.90)	17.10 (1.37–32.83)
Unadjusted HR (95% CI)	Ref	**0.48 (0.26–0.89)**	**0.51 (0.33–0.79)**	0.98 (0.51–1.87)
*Adjusted HR (95% CI)	Ref	0.52 (0.27–1.02)	**0.56 (0.35–0.90)**	1.22 (0.61–2.44)
Stroke				
No. of patients	7	0	1	0
Event rate, per 100 person-years	1.11 (1.07–1.16)	0.0 (0.0–0.0)	0.39 (0.33–0.46)	0.0 (0.0–0.0)
Unadjusted HR (95% CI)	Ref	N/A	0.36 (0.05–2.96)	N/A
*Adjusted HR (95% CI)	Ref	N/A	0.13 (0.00–4.30)	N/A

^a^ Data are presented as number or hazard ratio (95% CI).

^b^ The primary outcome was a composite of all-cause death, hospitalization for heart failure or stroke.

Abbreviations as in Table 3. HRs (bold) are statistically significant (*p*-value <0.05). * = Adjusted for covariates including ages, sex, body mass index, smoking, alcohol, NYHA III/IV, hypertension, diabetes; SBP, heart rate, myocardial infarction, coronary artery disease, atrial fibrillation, stroke, potassium, creatintine, eGFR, LVEF, ACEI/ARB, beta-blockers, diuretics, statin.

### Renal function and outcomes

Overall, when we classified patients according to categories defined by their CKD stages, there were 102 (26%) patients with an eGFR of 45–60 mL/min/1.73 m^2^ (corresponding CKD stages IIIA), 119 (31%) patients with an eGFR of 30–45 mL/min/1.73 m^2^ (CKD stage IIIB), and 166 (43%) patients with an eGFR of < 30 mL/min/1.73 m^2^ (CKD stage IV and V). Compared with those in higher eGFR categories, patients in the lowest eGFR category (< 30 mL/min/1.73 m^2^) was associated with a higher risk of worse outcome events. With eGFR of 45–60 mL/min/1.73 m^2^ as reference, patients with severe renal dysfunction (eGFR< 30 mL/min/1.73 m^2^) showed a higher risk of primary outcome, all-cause death and hyperkalemia [unadjusted HR (95% CI) for primary outcome: 1.86 (1.29–2.68); unadjusted HR (95% CI) for all-cause death: 4.09 (1.59–10.53); unadjusted HR (95% CI) for hyperkalemia: 3.89 (1.15–13.20)]. No significant difference was noted between baseline eGFR and the risk of HF hospitalization or stroke (Table 5).

**TABLE 5:** Primary outcomes events in patients categorized according to the dose of spironolactone (Risk estimates calculated using > 40 mg as reference)^a^.

	Dose of spironolactone			
Events	No use *n* = 233	< 40 mg *n* = 41	40 mg *n* = 91	> 40 mg *n* = 22
Primary outcome events^b^				
No. of patients	135	12	31	12
Event rate, per 100 person-years	21.45 (16.18–26.72)	10.16 (0.91–19.41)	12.14 (5.43–18.85)	20.52 (3.64–37.40)
Unadjusted HR (95% CI)	1.01 (0.56–1.82)	**0.41 (0.18–0.91)**	**0.50 (0.26–0.98)**	Ref
*Adjusted HR (95% CI)	0.87 (0.46–1.65)	**0.38 (0.16–0.86)**	**0.43 (0.22–0.86)**	Ref
All cause death				
No. of patients	39	2	10	3
Event rate, per 100 person-years	6.20 (3.10–9.29)	1.69 (1.40–2.02)	3.92 (3.71–4.13)	5.13 (4.19–6.16)
Unadjusted HR (95% CI)	1.20 (0.37–3.87)	0.33 (0.06–1.99)	0.76 (0.21–2.77)	Ref
*Adjusted HR (95% CI)	0.99 (0.27–3.60)	0.30 (0.05–1.93)	0.63 (0.16–2.46)	Ref
Re-hospitalization for heart failure				
No. of patients	111	11	25	10
Event rate, per 100 person-years	17.64 (12.74–22.53)	9.32 (0.42–18.21)	9.79 (3.69–15.90)	17.10 (1.37–32.83)
Unadjusted HR (95% CI)	1.02 (0.53–1.95)	0.49 (0.21–1.15)	0.52 (0.25–1.08)	Ref
*Adjusted HR (95% CI)	0.82 (0.41–1.65)	0.43 (0.18–1.05)	**0.46 (0.22–0.98)**	Ref
Stroke				
No. of patients	7	0	1	0
Event rate, per 100 person-years	1.11 (1.07–1.16)	0.0 (0.0–0.0)	0.39 (0.33–0.46)	0.0 (0.0–0.0)
Unadjusted HR (95% CI)	N/A	N/A	N/A	Ref
*Adjusted HR (95% CI)	N/A	N/A	N/A	Ref

^a^ Data are presented as number or hazard ratio (95% CI).

^b^ The primary outcome was a composite of all-cause death, hospitalization for heart failure or stroke. Abbreviations as in Table 3.

HRs (bold) are statistically significant (*p*-value <0.05). * = Adjusted for covariates including ages, sex, body mass index, smoking, alcohol, NYHA III/IV, hypertension, diabetes; SBP, heart rate, myocardial infarction, coronary artery disease, atrial fibrillation, stroke, potassium, creatintine, eGFR, LVEF, ACEI/ARB, beta-blockers, diuretics, statin.

Furthermore, we examined the effect of spironolactone across all eGFR spectrum. Table 6 showed the result of efficacy of spironolactone in each eGFR category, the risk of primary outcome, as well as the absolute risk difference, were consistent across full range of eGFR [adjusted HR (95% CI) for total population: 0.53 (0.39–0.73); *p* for interaction: 0.17; absolute risk difference for total population: 9.01%], which indicated that the efficacy of spironolactone remained consistent across the full range of eGFR.

**TABLE 6:** Incidence rates and hazard ratios for cardiovascular outcomes by eGFR category.

	eGFR categories, mL/min/1.73m^2^								
	45–60 (*n* = 102, 26%)			30–45 (*n* = 119, 31%)			<30 (*n* = 166, 43%)		
Clinical outcomes	Incidence Rate	HR (95% CI)	*p*-value	Incidence Rate	HR (95% CI)	*p*-value	Incidence Rate	HR (95% CI)	*p*-value
Primary outcome	13.69	Ref	Ref	15.87	1.12 (0.74–1.68)	0.592	22.35	1.86 (1.29–2.68)	0.001
All-cause death	1.67	Ref	Ref	5.80	3.43 (1.28–9.18)	0.014	6.91	4.09 (1.59–10.53)	0.004
HF re-hospitalization	12.35	Ref	Ref	13.43	1.03 (0.66–1.59)	0.903	17.51	1.47 (0.99–2.18)	0.056
Stroke	1.00	Ref	Ref	0.00	N/A	0.965	1.15	1.02 (0.25–4.28)	0.975
Adverse event Hyperkalemia	1.00	Ref	Ref	2.14	2.05 (0.53–7.94)	0.297	4.15	3.89 (1.15–13.20)	0.029

HF, heart failure; other abbreviations as in Tables 1, 3.

### Safety

With respect to the safety, there were 15 serious adverse events in no use of spironolactone group and 15 in the spironolactone group (2.38 per 100 person-years and 3.47 per 100 person-years, respectively). Hyperkalemia occurred in 13 (8.4%) and 15 (6.4%) patients in the spironolactone group and no use of spironolactone group (HR: 1.76; 95% CI: 0.73–4.28; *p* = 0.21).

Regarding the dose interaction, patients taking 40 mg spironolactone showed the lowest incidence rate of hyperkalemia when compared to the < 40 mg and > 40 mg groups (*p* = 0.001). Additionally, the incidence rates of hyperkalemia were 2-fold higher in patients taking high-dose of spironolactone (> 40 mg) compared with those taking low-dose (≤ 40 mg) spironolactone (5.13 per 100 patient-years vs. 2.68 per 100 patient-years). However, with no use as reference, there was no significant difference between three groups [adjusted HR (95% CI):1.58 (0.62–4.00) with ≤ 40 mg group, *p* = 0.34; 3.25 (0.77–13.78) with > 40 mg group, *p* = 0.11)] (Table 7). The change of serum potassium before and after taking spironolactone also showed no significant difference between low-dose and high-dose group (0.68 mmol/L ± 0.69 mmol/L vs. 0.53 mmol/L ± 0.68 mmol/L, *p* = 0.341). Moreover, after grouped by eGFR, in spironolactone group, hyperkalemia occurred in 3 (6.4%), 6 (10.2%) and 4 (8.3%) in stage IIIA, IIIB and IV/V, respectively. There was no statistically significant difference among the three groups (*p* = 0.934). While, among patients not taking spironolactone, 14 (11.9%) patients in stage IV and V eventually developed hyperkalemia which showed a significant difference (*p* = 0.002). In addition, in spironolactone group, there were two patients in low-dose group occurred permanent drug discontinuation (one for hyperkalemia and one for unknown reason). Table 8.

**TABLE 7:** Impact of spironolactone on clinical outcomes by estimated glomerular filtration rate (eGFR) category.

	Efficacy (all-cause death, HF re-hospitalization, or Stroke)			
eGFR (ml/min/1.73m^2^)	Incidence rate (per 100py)		Treatment effect	
	No use (*n* = 233)	Spironolactone (*n* = 154)	Hazard Ratio (95%CI)	Absolute risk difference
Total population	21.45	12.74	**0.53 (0.39–0.73)**	9.01%
45–60 (*n* = 102)	16.18	10.81	0.65 (0.27–1.56)	5.37%
30–45 (*n* = 119)	22.91	9.38	**0.22 (0.10–0.48)**	13.53%
< 30 (*n* = 166)	23.43	19.58	0.64 (0.35–1.15)	3.85%
InteractionP-value			0.17	

Py, patient-year; other abbreviations as in Tables 3, 6.

HRs (bold) are statistically significant (*p*-value <0.05).

**TABLE 8:** Association between spironolactone and safety outcomes.

	Safety (Hyperkalemia)	
Category	Incidence Rate (per 100py)	Hazard Ratio (95%CI)
With or without spironolactone (With no use as reference)		
No use	2.38	Ref
Spironolactone	3.01	1.76 (0.73–4.28)
Dose of spironolactone (With no use as reference)		
No use	2.38	Ref
≤40 mg	2.68	1.58 (0.62–4.00)
>40 mg	5.13	3.25 (0.77–13.78)

Abbreviations as in Tables 3, 7.

## Discussion

In this single-center analysis of patients with HFpEF and CKD, we explored the dose of spironolactone and their association with prognosis. We found that patients taking spironolactone were at significantly lower risk of primary outcomes, as well as HF hospitalization. Notably, patients receiving a low-dose of spironolactone (< 40 mg and 40 mg) had a better clinical outcome, while those taking > 40 mg spironolactone per day did not benefit from spironolactone use. In addition, the adding spironolactone to existing therapy in these patients did not significantly increased the incidence of the hyperkalemia. When it came to dose comparisons, patients taking 40 mg spironolactone had a lower incidence rate of hyperkalemia than the other two groups, while no significant difference was observed between high-dose group and low-dose group. Although the incidence of adverse events including hyperkalemia and serum potassium changes appeared to be consistent between the high-dose and low-dose groups, the low-dose group (≤ 40 mg) showed a better efficacy and risk balance (especially 40 mg group) compared to the high-dose group, suggesting that spironolactone doses ≤ 40 mg/day (around 20 mg/day–40 mg/day) may be used in patients with HFpEF and CKD.

The RALES and TOPCAT trials had demonstrated the benefits of spironolactone in HFrEF and HFpEF, respectively (Barr, 1996; Pitt et al., 2014). Based on the findings from two *post hoc* analyses of “Americas-population” in TOPCAT, spironolactone could reduce the risk of HF hospitalizations with consistent efficacy across the entire eGFR range (except eGFR< 30 ml/min/1.73 m^2^), although patients with poor renal function require close surveillance (Pfeffer et al., 2015; Beldhuis et al., 2019), which suggested that spironolactone can produce a marked effect in HFpEF patients with CKD and therefore recommended as Class IIB by 2022AHA Guidelines (Heidenreich et al., 2022). In the present study, use of spironolactone effectively reduced adverse cardiovascular events in HFpEF patients with CKD, and most notably in reducing the HF hospitalization, which is consistent with previous trial findings. In EMPEROR-Preserved trial, the 21% lower relative risk of primary outcome was mainly related to a 29% lower risk of hospitalization for heart failure (Anker et al., 2021). Also, a meta-analysis showed no benefit in the risk of all-cause mortality or cardiovascular death for six therapeutic drugs, including SGLT2i, ARNI and mineralcorticoid receptor antagonist (MRA). Compared with placebo, SGLT2i, ARNI and MRA were related to the significant reduction of HF hospitalization risk, among which SGLT2i had the most significant effect (Xiang et al., 2022). Moreover, the results of the DELIVER study demonstrated that dagliflozin reduces the risk of cardiovascular death or worsening heart failure in HFpEF patients independent of baseline renal function (Solomon et al., 2022), our study also included patients with eGFR< 30 ml/min/1.73 m^2^, and confirmed the consistency of the efficacy of spironolactone in this population, further demonstrating the benefit of spironolactone in patients with HFpEF and CKD. Unlike spironolactone, SGLT2i can also delay the decline in eGFR, achieving a cardiorenal protection effect (Anker et al., 2021; Solomon et al., 2022).

While, the use of spironolactone is often accompanied by the occurrence of hyperkalemia. The risk of hyperkalemia during spironolactone treatment of HFrEF is well documented (Pitt et al., 1999; Wrenger et al., 2003; Juurlink et al., 2004; Wei et al., 2010), independent risk factors for hyperkalemia such as assignment to spironolactone, lower eGFR level, higher baseline potassium and diabetes had also been described in the EMPHASIS-HF and RALES trials (Shah et al., 2005; Zannad et al., 2011; Eschalier et al., 2013; Vardeny et al., 2014), of which higher doses of spironolactone expectedly led to higher rates of hyperkalemia. Therefore, for the use of spironolactone in HFrEF, the guidelines have clear dosage instructions (25 mg/d–50 mg/d) (McDonagh et al., 2021). The current guidelines, however, have no accurate recommendation for the use of spironolactone in HFpEF patients. Two *post hoc* analyses of TOPCAT had clarified that the HFpEF population with poorer renal function had a higher incidence of adverse events such as hyperkalemia during the use of spironolactone (Desai et al., 2018; Beldhuis et al., 2019). Our study also confirmed that patients with lowest eGFR category (< 30 ml/min/1.73 m^2^) assigned to spironolactone had higher rates of hyperkalemia (10.8%). Thus, the use of spironolactone, including dose titration, requires closer monitoring in patients with HFpEF and CKD.

Previous large RCTs of spironolactone in patients with HFpEF had not performed the efficacy comparison between high and low doses (Edelmann et al., 2013; Pitt et al., 2014; Kosmala et al., 2016). So far, the best available evidence comes from the secondary analysis of TOPCAT, which suggested high-risk HFpEF patients may use low-dose spironolactone (< 25 mg/day) to obtain the benefits (Ferreira et al., 2020). However, the analysis did not make a direct comparison between the high and low dose groups. Our outcome analysis partially verifies the previous observation, when compared with patients not taking spironolactone, we found lower CV risk in patients taking low-dose spironolactone (≤ 40 mg) as compared to those not taking spironolactone. Vardeny et al. (2012) found that low-dose spironolactone treatment (≤ 40 mg/day) of patients with CKD tended to reduce the incidence of all-cause death and cardiogenic re-admission. Moreover, Matsumoto et al. (2014), Saudan et al. (2003) demonstrated that low-dose spironolactone effective in reducing cardiovascular mortality and was not associated with an increased frequency of hyperkalemia in hemodialysis patients. Thus, low-dose spironolactone may be considered beneficial for both left ventricular function and renal function. More than that, our data included patients with eGFR< 30 mL/min/1.73 m^2^ and on dialysis, and their baseline serum potassium < 4.5 mmol/L, which amplifies and extends the range of populations benefiting from low-dose spironolactone compared to previous analysis. Furthermore, when directly compare “high vs. low” doses of spironolactone, patients in low-dose group had the better long-term prognosis. Base on our data, among patients taking 40 mg and < 40 mg spironolactone, they were associated with a 50% and 57% lower risk of primary composite outcome. In contrast, patients taking high-dose spironolactone showed no significant benefit when compared with those not taking spironolactone. In the ATHENA-HF trial, patients used high-dose spironolactone (100 mg/day) did not improve the primary or secondary efficacy end points as to compared with usual care in acute heart failure patients (Butler et al., 2017), however, the duration of this high-dose treatment was relatively short (only 96 h or till discharge), the long-term practical effects cannot be seen. In our study, though the high-dose group had one-sixth the number of people in the low-dose group, the incidence of hyperkalemia was twice as high as the low-dose group, so the high adverse event rate may offset the benefit of spironolactone. While the relatively small sample size of high-dose group (22 patients) encouraged for further large research.

**FIGURE 3:**
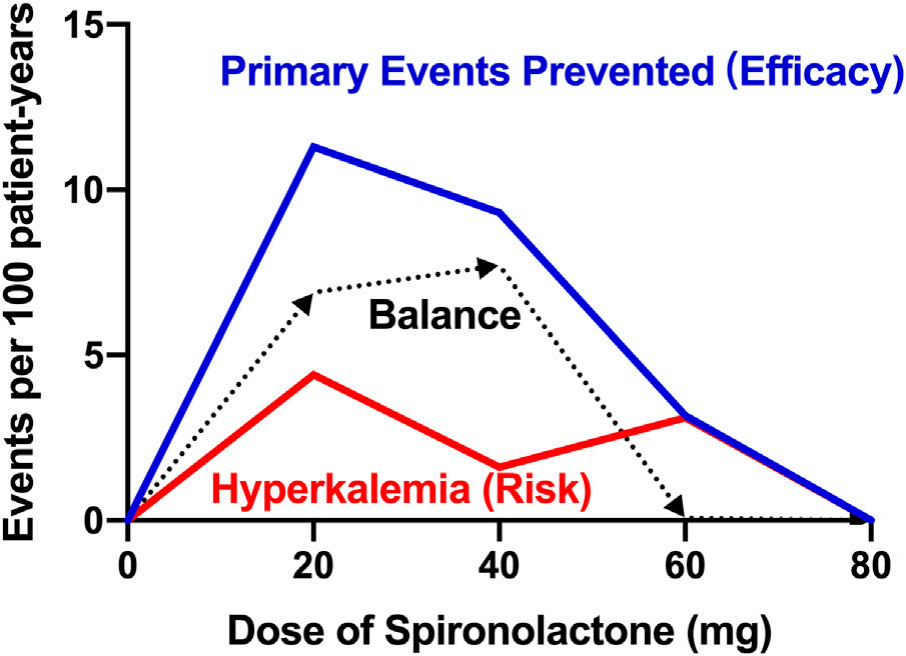
Title: Balance of efficacy and risk of spironolactone Legend: Balance difference between efficacy and safety of different doses of spironolactone. Efficacy is defined as the treatment effect on the prevented primary endpoint (all-cause death, heart failure hospitalization, or stroke). Risk is defined as the risk of hyperkalemia (serum potassium > 5.5 mmol/L). Balance is defined as the difference value between efficacy and risk. The higher the balance value, the better the comprehensive effect of the dose.

In contrast to the treatment effect, the data depicted in our report show that there is no significant difference in the incidence of hyperkalemia between patients taking and not taking spironolactone. Patients taking high-dose spironolactone in ATHENA-HF trial were well tolerated and did not show a high incidence of hyperkalemia (Butler et al., 2017). Based on the low occurrence of hyperkalemia in both groups, on the one hand, the risk of spironolactone-induced hyperkalemia may be clinically overestimated, on the other hand, patients not taking spironolactone had poorer baseline renal function and more than half of these patients were on CKD stage IV, which may have contributed to the high incidence of hyperkalemia during follow-up period. Besides, according to our data, among patients taking low-dose spironolactone (≤ 40 mg), we could expect to prevent 9.9 occurrences of primary composite event, but cause 0.3 occurrences of hyperkalemia for every 100 patients. However, for those taking high-dose spironolactone (> 40 mg), the preventable event and induced adverse event numbers were 0.9 primary outcomes and 3.1 hyperkalemia events, respectively. Moreover, based on the relationship between efficacy and risk, the Figure 3 shows the balance between the various dose groups, which emphasizes that the use of low-dose of spironolactone (≤ 40 mg) is able to obtain a better clinical outcome while reducing risk of adverse events. Overall, these findings support the use of spironolactone and its optimal dose in HFpEF patients with CKD, and also emphasize the closer laboratory monitoring of serum potassium and renal function changes in advanced CKD patients during up-titration period.

## Limitations

Some limitations of our study should be considered. First, this study was a single-center, clinical, retrospective cohort study, which limited power to examine the effects of treatment. We cannot completely exclude the potential effects of confounders, and given the sample size of present study was relatively small, this study should be considered as exploratory, further prospective adequately powered randomized trials are needed before this conclusion is applied to clinical practice. Second, our center lacks echocardiographic indicators such as E/e’ ratio, septal and lateral e’ value, thus the diagnostic definition for HFpEF is relatively imperfect. Third, we skipped the spironolactone titration process, mainly focused on the long-term dose maintenance period, and performed the analysis on this basis. It may lead us to ignore some events that occur in patients during up-titration. In addition, we paid less attention to the worsening renal function and gynecomastia and other adverse events caused by spironolactone. Finally, we intended to analyze the effect of spironolactone on dialysis patients, but due to the small number of events in dialysis patients, however, due to the fewer incidence rates, the further exploratory studies will be required.

## Conclusion

The findings of our real-world study revealed that use of spironolactone can reduce the risk of cardiovascular events in HFpEF patients with CKD. Low-dose spironolactone (≤ 40 mg) was associated with improvement in the primary outcome and the intervention was relatively safe. Considering the balance of efficacy and risk, we may recommend ≤ 40 mg as the optimal dose of spironolactone for patients with HFpEF and CKD. Non-etheless, larger randomized clinical trials are needed to further confirm the efficacy and safety of spironolactone in HFpEF combined with CKD patients.

## Data Availability

The raw data supporting the conclusion of this article will be made available by the authors, without undue reservation.

## Glossary

**HFpEF**: heart failure with preserved ejection
**CKD**: chronic kidney disease
**HF**: heart failure
HR: hazard ratio
**95% CI**: 95% confidence interval
**EMPEROR-Preserve**: Empagliflozin in Heart Failure with a Preserved Ejection Fraction
**DELIVER**: Dapagliflozin in Heart Failure with Mildly Reduced or Preserved Ejection Fraction
**SGLT2i**: sodium-dependent glucose transporters 2 inhibitor
**TOPCAT**: Treatment of Preserved Cardiac Function Heart Failure With an Aldosterone Antagonist
**ICD**: International Classification of Diseases
**EMR**: Electronic-Medical-Records System
**CKD-EPI**: Chronic Kidney Disease Epidemiology Collaboration equation
**EF**: ejection fraction
**LVEF**: left ventricle ejection fraction
**ESC**: European Society of Cardiology
**AHA**: American Heart Association
**I-Preserve**: Irbesartan in patients with heart failure and preserved ejection fraction
**PARAGON-HF**: Angiotensin Receptor Neprilysin Inhibition in Heart Failure With Preserved Ejection Fraction
**NYHA**: New York Heart Association
**AF**: atrial fibrillation
**LVMI**: left ventricle mass index
**NSAID**: nonsteroidal anti-inflammatory drugs
**SD**: standard deviation
**IQR**: interquartile range
**BMI**: body mass index
**ACEI**: angiotensin-converting–enzyme inhibitors
**ARB**: angiotensin-II–receptor blockers
**RCT**: randomized controlled trial
**RALES**: The effect of spironolactone on morbidity and mortality in patients with severe heart failure.
**EMPHASIS-HF**: Eplerenone in patients with systolic heart failure and mild symptoms
**CV**: cardiovascular
**ATHENA-HF**: Efficacy and safety of spironolactone in acute heart failure
